# Are the Immune Properties of Mesenchymal Stem Cells from Wharton’s Jelly Maintained during Chondrogenic Differentiation?

**DOI:** 10.3390/jcm9020423

**Published:** 2020-02-04

**Authors:** Charlotte Voisin, Ghislaine Cauchois, Loïc Reppel, Caroline Laroye, Laetitia Louarn, Chantal Schenowitz, Paulin Sonon, Isabelle Poras, Valentine Wang, Edgardo D. Carosella, Nadia Benkirane-Jessel, Philippe Moreau, Nathalie Rouas-Freiss, Danièle Bensoussan, Céline Huselstein

**Affiliations:** 1 UMR 7365 CNRS-Université de Lorraine, Ingénierie Moléculaire et Physiopathologie Articulaire (IMoPA), Biopôle de l’Université de Lorraine, Campus brabois-santé, Faculté de Médecine, 9 Avenue de la Forêt de Haye, BP 184, 54500 Vandoeuvre-lès-nancy, France; ghislaine.cauchois@univ-lorraine.fr (G.C.); loic.reppel@univ-lorraine.fr (L.R.); c.laroye@chru-nancy.fr (C.L.); valentinewang21@gmail.com (V.W.); d.bensoussan@chru-nancy.fr (D.B.); Celine.Huselstein@univ-lorraine.fr (C.H.); 2 UMS2008 IBSLor, Campus brabois-santé, 9 Avenue de la Forêt de Haye, BP20199, 54500 Vandoeuvre-lès-nancy, France; 3 CHRU de Nancy, Unité de Thérapie Cellulaire Banque de Tissus, 54500 Vandœuvre-lès-Nancy, France; 4 CEA, DRF-Institut François Jacob, Service de Recherches en Hémato-Immunologie, Hopital Saint-Louis, 75010 Paris, France; laetitialouarn@gmail.com (L.L.); Chantal.Schenowitz@cea.fr (C.S.); paulinsonon@gmail.com (P.S.); isabelle.poras@cea.fr (I.P.); edgardo.carosella@cea.fr (E.D.C.); philippe.moreau@cea.fr (P.M.); nathalie.rouas-freiss@cea.fr (N.R.-F.); 5 Université de Paris, CEA, U976 HIPI Unit (Human Immunology, Physiopathology, Immunotherapy), Institut de Recherche Saint-Louis, 75010 Paris, France; 6 INSERM-UNISTRA UMR1260, Regenerative Nanomedicine laboratory, Faculté de Médecine, FMTS, Strasbourg CEDEX F-67085, France; nadia.jessel@inserm.fr

**Keywords:** mesenchymal stem/stromal cells, immunomodulation, cell differentiation, allogeneic context, Advanced Therapy Medicinal Product

## Abstract

Background: Umbilical mesenchymal stem/stromal cells (MSCs), and especially those derived from Wharton’s jelly (WJ), are a promising engineering tool for tissue repair in an allogeneic context. This is due to their differentiation capacity and immunological properties, like their immunomodulatory potential and paracrine activity. Hence, these cells may be considered an Advanced Therapy Medicinal Product (ATMP). The purpose of this work was to differentiate MSCs from WJ (WJ-MSCs) into chondrocytes using a scaffold and to evaluate, in vitro, the immunomodulatory capacities of WJ-MSCs in an allogeneic and inflammatory context, mimicked by IFN-γ and TNF-α priming during the chondrogenic differentiation. Methods: Scaffolds were made from hydrogel composed by alginate enriched in hyaluronic acid (Alg/HA). Chondrogenic differentiation, immunological function, phenotype expression, but also secreted soluble factors were the different parameters followed during 28 days of culture. Results: During chondrocyte differentiation, even in an allogeneic context, WJ-MSCs remained unable to establish the immunological synapse or to induce T cell alloproliferation. Moreover, interestingly, paracrine activity and functional immunomodulation were maintained during cell differentiation. Conclusion: These results show that WJ-MSCs remained hypoimmunogenic and retained immunomodulatory properties even when they had undergone chondrocyte differentiation.

## 1. Introduction

For several years, regenerative medicine and tissue engineering (TE) have been hot fields in basic and clinical research. They rely on the replacement, repair, or restoration of injured tissue using scaffolds associated with stem cells [1]. Human mesenchymal stem/stromal cells (MSCs) seem very promising in this area for regenerating non-functional tissues because of their differentiation properties (adipocytes, chondrocytes, or osteocytes) [2,3,4].

First isolated from bone marrow in 1976 by Friedenstein, MSCs are described as fibroblastic cells of a mesodermal origin. Nowadays, MSCs can be isolated from a variety of other tissues including periosteum, trabecular bone, adipose tissue, synovial membrane, skin, or skeletal muscles [3]. They can also be found in fetal tissues like the placenta or the conjunctive tissue of the umbilical cord matrix, named Wharton’s jelly (WJ) [5]. Although Wharton’s jelly mesenchymal stem/stromal cells (WJ-MSCs) share similar characteristics with MSCs from adult tissues, they have higher proliferation potential, can differentiate in a large number of cells [6], and seem more immature based on their immunological properties. All these advantages make WJ a virtually inexhaustible source of stem cells, especially for allogeneic TE therapies. Furthermore, their sampling is neither invasive nor iatrogenic compared to adult tissue.

For degenerative osteoarthritic (OA) diseases, a therapeutic option could be the regeneration of cartilage defects. Thus, TE is a promising alternative that involves the development of biological substitutes composed of cells embedded into natural or synthetic scaffolds. Cartilage TE is based on three main aspects: the cell source, a three-dimensional (3D) framework that mimics the physiological environment of the tissue, and, finally, complete functionalization to reach the expected biological functions [7]. Regarding the cell source, MSCs, thanks to their ability to differentiate into various cells, are good candidates to differentiate in a 3D environment [8]. Indeed, a 3D environment is necessary to create an effective chondro-differentiation, most often using scaffolds. These scaffolds should be biodegradable or bioresorbable, biocompliant, non-immunogenic, and non-toxic for clinical use. Many types of scaffold can be built, like hydrogel, sponge, or mesh. They can be natural (alginate, hyaluronic acid, collagen, and fibrin) or synthetic (poly lactic-co-glycolic acid (PLGA) or polycaprolactone (PCL)). In this work, we chose to use alginate (Alg), a monophasic biomaterial that seems to provide an advantageous environment for chondrogenic differentiation. Alginate is a polysaccharide with a chemical structure composed of mannuronic acid (M) and guluronic acid (G); it can be mixed with an extracellular matrix component like hyaluronic acid (HA), a cartilage matrix component [9,10]. Functionalization may be achieved by adding growth factors such as Type 2 Bone Morphogenic Protein (BMP2) or Transforming Growth Factor-β (TGF- β) [9,10,11,12].

More recently, MSCs have been studied for their immunomodulatory properties. These cells can escape from the recipient’s immune system and modulate it, allowing their use in an allogeneic context. The immune properties of MSCs rely on three main mechanisms: the arrest of the cell cycle of immune cells at the G1 phase, a direct interaction of MSCs with immune cells, and a paracrine effect. They express neither major histocompatibility complex class II (MHC-II) nor the costimulatory molecules: CD80, 86, and 40, which are important molecules of the immunological synapse. Consequently, they are not recognized by CD4+ T lymphocytes and cannot activate T cells [13,14,15]. It was also reported that MSCs have anti-inflammatory properties in a pro-inflammatory context in the presence of Interferon-γ (IFN-γ). This immunosuppressive activity is related to the expression of a strong immune checkpoint molecule, Programmed Death-Ligand 1 (PD-L1) named CD274 or even B7-H1. When these ligands are expressed on the cell surface, they bind to the PD-1 receptor on T cells and allow their inactivation. Immunomodulation has also been linked to other molecules such as Indoleamine 2,3 dioxygenase (IDO). This enzyme (IDO) induces regulatory T cells by depleting tryptophan from the extracellular environment, secondarily preventing proliferation of T cells [16].

A non-classic HLA class I molecule, i.e., HLA-G (a molecule involved in maternofetal tolerance) has been implicated in MSC-mediated immunomodulation by direct (membrane) or indirect (soluble) contact [17]. HLA-G molecules expressed by MSCs fulfill an important function since the blockade of HLA-G with neutralizing antibodies was shown to reverse the ability of MSC to (i) generate the in vitro expansion of CD4+CD25+ FoxP3+ regulatory T cells, (ii) inhibit the alloproliferative T cell response, and (iii) suppress the cytotoxic function of Natural Killer (NK) cells. These results show that HLA-G molecules actively contribute to the immunomodulatory properties exhibited by MSCs [18]. MSCs can secrete many other soluble factors, such as growth factors, cytokines, chemokines or immune molecules, to create an immunosuppressive environment. These soluble factors will behave as mediators suppressing the proliferation of NK or T cells, especially with Prostaglandin E2 (PGE-2), Transforming Growth Factor-β (TGF- β) or Hepatocyte Growth Factor (HGF). Moreover, several cytokines, like Interleukin 6 and 10 (IL-6 and IL-10), have also been involved in this mechanism, to prevent inflammation and the differentiation of dendritic cells. Vascular Endothelial Growth Factor (VEGF) was suggested to act in anti-apoptosis and inflammation modulation [15,19]. Other soluble molecules and chemokines may also contribute to immunosuppression, among them PD-L1, which can be secreted to induce tolerance [20].

In this study, we designed a biomaterial using MSCs from Wharton’s jelly as an Advanced Therapy Medicinal Product (ATMP). However, the persistence of the immune properties of WJ-MSCs during chondrogenic differentiation remains unknown, especially when WJ-MSCs are embedded into a scaffold and submitted to an allogeneic context. Therefore, the aim of this study was to investigate immunomodulatory properties of this innovative product manufactured with WJ-MSCs encapsulated in an alginate-based hydrogel enriched with hyaluronic acid (Alg/HA) to guide chondrocyte differentiation.

## 2. Experimental Section

### 2.1. Human Umbilical Cord Collection, Wharton Jelly-Mesenchymal Stem Cell Isolation, Expansion, and Conservation

Umbilical cords (*n* = 7, named C1 to C7) were collected at the Maternity Hospital of Nancy. This collection was approved by the Nancy Hospital ethics committee and French Ministry of Research (No. DC-2014-2114). All the productions of WJ-MSCs were performed in clinical grade conditions with a complete medium containing α-MEM culture medium (Macopharma, Mouvaux, France) enriched with 5% Platelet lysate (Macopharma, Mouvaux, France) and according to good manufacturing practices. After brief immersion of the umbilical cord in an antibiotic-antifungal solution, it was cut into thin sections and placed in small flasks (Dutscher, France) containing a complete medium at 37 °C and 5% CO_2_ under hypoxia conditions (2% of O_2_). The medium was changed after 4 to 5 days. After 10 days, when cell migration was observed, cord pieces were removed and the medium changed. At the end of Passage 0 (P0), when 80–90% subconfluence was reached, WJ-MSCs were rinsed with Phosphate Buffered Saline (PBS, Macopharma, Mouvaux, France), detached by trypsin action (TrypLe, Fischer Scientific, France) and washed by centrifugation. The cells were seeded in Passage 1 (P1) with a seeding kit (Macopharma, Mouvaux, France) in CellStack culture containing two chambers (Macopharma, Mouvaux, France) at a density of 1000 cells per cm^2^. At the end of Passage 2 (P2), WJ-MSCs were frozen in a cryopreservation solution (80% albumin and 20% DMSO) and stored in vapor phase nitrogen. For thawing, WJ-MSCs were quickly thawed and washed before being cultivated at 37 °C under hypoxic conditions, as previously described. After reaching 80–90% subconfluence, WJ-MSCs were harvested to be seeded in Alg-based hydrogels. This step corresponded to Day 0 (D0) of the chondro-differentiation process.

### 2.2. Quality Controls

#### 2.2.1. Donor Serology and MSC Production Sterility

Umbilical cords were tested for HIV, HBV and HCV via a serological and nucleic acid test (NAT), and for HTLV, Syphilis, EBV, CMV, and toxoplasmosis via serological tests. Cells were tested during expansion, at each medium change, trypsinization stage, and before cryopreservation with BacT/Alert device (BioMérieux, Marcy-l’Etoile, France), for aerobic and anaerobic bacteria absence.

#### 2.2.2. MSC Characterization

Before cryopreservation, cells were detached with TrypLe and counted by trypan blue dye exclusion. Immunophenotype and viability of WJ-MSCs were performed using flow cytometry. Cells were stained with antibodies from the Human MSC Analysis Kit (BD Stemflow™, BD Biosciences, San Diego, CA, USA), including the MSC positive cocktail (CD90-FITC (Fluorescein isothiocyanate), CD105-PerCP-Cy™5.5 (Peridinin Chlorophyll Protein-Cyanin), CD73-APC (Allophycocyanine), CD44-PE (phycoerythrin)) and the MSC negative cocktail (CD45-PE, CD34-PE, CD11b-PE, CD19-PE and HLA-DR-PE). Seven-Amino-Actinomycin D (7-AAD) (BD Biosciences, San Jose, CA, USA) staining was also performed as a viability assessment.

The clonogenic capacity was evaluated using colony-forming unit fibroblast (CFU-F) assays. MSCs were seeded in T25 flasks, at 10 and 20 cells/cm^2^. They had been cultured for 10 days in the previously described complete medium. Then, they were washed with PBS, fixed with ethanol, stained with a Giemsa solution (Sigma, St. Louis, MO, USA), and rinsed with water. A Colony-forming unit fibroblast of more than 50 cells were scored and the percentage of clonogenicity was calculated based on the ratio of the number of colonies counted and the number of cells seeded.

Adipogenic and osteogenic differentiations were performed according to the manufacturer’s instructions (Differentiation Media BulletKits, Lonza, Switzerland). Calcium mineralization was assessed by coloration with Alizarin Red (Sigma, St. Louis, MO, USA), and fluorescent staining was performed with AdipoRed™ (Lonza, Basel, Switzerland) to detect lipid droplets, which were observed by confocal microscopy.

MSC karyotype was performed by the Genetics Laboratory of Nancy Hospital. Mitoses were blocked briefly in the metaphase stage by colchicine, and then subjected to a hypotonic and fixed shock. Standard cytogenetic analysis was performed in quinacrinebanding (QFQ banding).

### 2.3. Construction of Scaffolds Seeded with WJ-MSCs for Chondrogenic Differentiation

After the detachment of trypsin-EDTA (0.025%) at P3, WJ-MSCs were washed and seeded, according to previous studies [11,21,22], in a solution of GMP grade alginate (Novamatrix, Sandvika, Norway) enriched in ultrapure hyaluronic acid (Novamatrix, Sandvika, Norway) in a ratio of 4:1 dissolved in 0.9% NaCl. The solution was delivered drop by drop and polymerized with CaCl_2_ in plastic inserts contained in a 24-well plate. Afterwards, scaffolds were put in contact with Dubelcco’s Modified Eagle Medium High Glucose (DMEM, Sigma, St. Louis, MO, USA) with 1 mM CaCl_2_. Ten percent clinical grade fetal bovine serum (GEHealthcare, HyClone, Fisher Scientific, Illkirch, France), 2mM glutamine, 100 IU/mL penicillin, 100 µg/mL streptomycin and 2.5 µg/mL amphotericin B were added to this medium. DMEM concentrate containing 100 µg/mL sodium pyruvate, 50 µg/mL ascorbic acid, 4 µg/mL L-proline, and 10 nM dexamethasone was also added to the medium. Scaffolds were incubated in hypoxic conditions (5% CO_2_ and 2% O_2_) at 37 °C; the medium was changed twice a week until Day 28 (D28) of the culture. During these days of differentiation, cells were called chondro-MSCs. After 14 (D14) and 28 (D28) days of culture in scaffolds, the cells were extracted from the hydrogel scaffolds using 55 mM sodium citrate (Sigma, St. Louis, MO, USA) and 50 mM EDTA solution (Merck, Darmstadt, Germany) for 5 min. After centrifugation (300 g, 5 min), flow cytometry, and Reverse Transcription Polymerase Chain Reaction (RT-PCR) analysis, mixed lymphocyte reactions were performed and compared with MSCs at the end of P3 of monolayer expansion (D0). 

### 2.4. IFN-γ/TNF-α Priming of WJ-MSCs during Monolayer Expansion or Chondro-MSCs in Alg/HA Scaffolds

To mimic the inflammatory environment, cells (WJ-MSCs and chondro-MSCs) were stimulated with INF-γ (20 ng/mL) and TNF-α (30 ng/mL) (Miltenyi Biotec, Bergisch Gladbach, Germany), which were added to the culture medium. This stimulation occurred 48 hours before sub-confluence for monolayer culture and before D14 and D28 of 3D culture.

### 2.5. Histology

Scaffolds were fixed in 4% paraformaldehyde (Sigma, France), 100mM sodium cacodylate trihydrate and 10 mM CaCl_2_ dissolved in distilled water. They were dehydrated in ethanol baths of different concentrations and included in paraffin. Five micrometer sections were made and stained with sirius red (Sigma, St. Louis, MO, USA) and alcian blue (Searle Diagnostic, High Wycombe, UK) to detect total collagen fibers and proteoglycans, respectively. Images were acquired with the microscope DMD108 using X20 magnification (Leica, Wetzlar, Germany).

### 2.6. Flow Cytometry Experiments

Immune phenotype (membrane and intracellular) as well as pericellular collagen synthesis were analyzed by flow cytometry at the end of monolayer expansion (prior to encapsulation in the hydrogel) and throughout the scaffold culture. Immunolabeling was performed under saturating conditions; antibodies were considered to bind to surface antigens through monovalent interaction. Cells removed from Alg-based scaffolds were incubated with PBS/Bovine Serum Albumin 0.5% (PBS/BSA) (Sigma, St. Louis, MO, USA) and stained for 30 min at room temperature with direct membrane antibodies: CD34-PE, CD73-APC (BD Biosciences, San Jose, CA, USA), CD40-PE, CD80-PE, CD86-FITC (Miltenyi Biotec, Bergisch Gladbach, Germany), CD45-FITC (Dako Agilent, Santa Clara, CA, USA), CD90-FITC, CD105-PE, CD166-PE (Beckman Coulter, Brea, CA, USA), HLA-DPDQDR-FITC (Sony Biotechnology, San Jose, CA, USA) and PD-L1-APC (eBiosciences, San Diego, CA, USA). We used intracellular staining in order to detect unsecreted soluble molecules localized in the cytoplasm (soluble HLA-G and IDO). The Cytofix/Cytoperm kit (BD Biosciences, San Jose, CA, USA) was used to permeabilize the membrane according to manufacturer’s instructions. Secretion was not blocked as we had not previously observed any difference with secretion blockage. Then, cells were stained for 30 min at room temperature with anti-HLA-G1-FITC (MEM-G9 clone), anti- C-terminal amino acid sequence (22-mer) of soluble HLA-G5 and HLA-G6 proteins-FITC (2A12 clone) (Exbio, Vestec, Czech Republic), and anti-IDO-Alexa Fluor 488 (R&D systems, Minneapolis, MN, USA) antibodies.

WJ-MSCs were incubated for 30 min with rabbit anti-human type I, II, and X collagen antibodies (1:20) (Abcam, Cambridge, UK). Afterwards, cells were washed with PBS-BSA and centrifuged before bieng incubated for 30 min with a secondary antibody, a goat anti-rabbit IgG Alexa Fluor 488 (1:20) (Molecular Probes, Eugen, OR, USA). Negative controls were performed to detect cell auto-fluorescence.

Viability was performed with the LIVE/DEAD kit (Invitrogen, Cergy Pontoise, France). Then, cells were analyzed with the Gallios flow cytometer (Beckman Coulter, Brea, CA, USA) with an acquisition of 10,000 events. The cytometer was calibrated daily with Flow-Check Pro fluorospheres (Beckman Coulter, Brea, CA, USA). Flow cytometry results were analyzed with KALUZA software (Beckman Coulter, Brea, CA, USA).

### 2.7. Enzyme-Linked Immunosorbent Assay (ELISA)

For Enzyme-Linked Immunosorbent Assay (ELISA), cells were cultivated in Passage 3, for monolayer (WJ-MSCs on Day 0), and in biomaterial (chondro-MSCs on Day 7, 14 and 28). Culture media were collected and centrifuged (1000 rpm/min, 5 min) before storage at –20°C. Soluble molecules secreted by WJ-MSCs and chondro-MSCs were measured by direct ELISA for PGE2, and sandwich ELISA for IL-6, IL-10, VEGF, TGF-β1, HGF, and PD-L1 (DuoSet Kit, R&D Systems, Minneapolis, MN, USA), as described by the manufacturer. The detection limits were 2500 to 39 pg/mL for PGE2, 600 to 9.38 pg/mL for IL-6, 2000 to 31.3 pg/mL for IL-10, VEGF and TGF-β, 8000 to 125 pg/mL for HGF, and, finally, 10,000 to 156 pg/mL for PD-L1. After media collection, the cells were counted. The supernatants were diluted except for IL-10, and ELISA values were corrected for total cell numbers.

### 2.8. Mixed Lymphocyte Reaction (MLR)

Mixed Lymphocyte Reaction was performed to test the immunogenic and immunoregulatory properties of WJ-MSCs and chondro-MSCs licensed or unlicensed with inflammatory cytokines. For this purpose, WJ-MSCs or chondro-MSCs pretreated or not pretreated with 20 ng/mL IFN-γ and 30 ng/mL TNF-α for 48 h were used as stimulating cells (immunogenicity assay) or third-party cells (immunosuppression assay) toward HLA-mismatched T cells presented into peripheral blood mononuclear cells (PBMC). PBMC were isolated from the blood of healthy volunteer donors from the French Blood Establishment (EFS, Saint-Louis Hospital, Paris, France) by density-gradient centrifugation over Ficoll-Paque PLUS (Sigma, St. Louis, MO, USA). Human B lymphoblastoid cell lines (LCL) 721.221 (ATCC®CRL1855™, Manassas, VA, USA), HLA class II-positive cells, were irradiated at a 75 Gy dose to be used either as a positive control in immunogenicity assay or as a stimulator cell in the immunosuppression assay. MLR experiments were set up in RPMI supplemented with 10% heat-inactivated fetal calf serum. In immunogenicity assays, PBMC (1 × 10^5^ cells/100 µL) and MSCs (from 0.5 × 10^5^ to 0.03 × 10^5^ cells/100 µL) or LCL (0.5 × 10^5^ cells/100 µL) were seeded in each well of 96-well plate. In the immunosuppression assay, PBMC (1 × 10^5^ cells/50 µL), LCL (0.5 × 10^5^ cells/50 µL), and MSCs (from 0.5 × 10^5^ to 0.03 × 10^5^ cells/100 µL) were seeded in each well of 96-well plate. In all assays, MLR lasted for 6 days at 37 °C in a humidified 5% CO_2_ and 21% O_2_ air atmosphere (normoxic conditions). On Day 5, [3H]-thymidine (1 mCi/well; PerkinElmer, Waltham, MA, USA) was added to each well and the solution was incubated for another 18 h. Cells were then harvested on filtermates A, and thymidine incorporation into the DNA was quantified using a β counter (Wallac 1450; Pharmacia, ALT, Amershan, UK). Allogeneic PBMC stimulated by LCL were considered maximal MLR (100% alloproliferation).

### 2.9. RT-PCR Analysis for HLA-G Expression and Regulation

Total RNA was extracted using miRNeasy Mini Kit (Qiagen, Hilden, Germany), according to the manufacturer’s instructions. A total of 1 to 5 µg of total RNA from each sample was reverse-transcribed to cDNA using oligo-(dT) 12–18 priming and Moloney Murine Leukemia Virus (M-MLV) (Invitrogen, Cergy Pontoise, France) at 42 °C for 1 hour. Duplex Real-time RT-PCR was performed in duplicates on 0.1–0.5 µg of cDNA in an ABI Prism 7000 SDS (Applied Biosystems, Courtaboeuf, France) with 40 amplification rounds using AmpliTaq™ DNA Polymerase with Buffer I (Applied Biosystems), predeveloped TaqMan GAPDH (Applied Biosystems) as an internal control, HLA-G specific primers that amplify exon 5 (forward: 5‘-CTGGTTGTCCTTGCAGCTGTAG; reverse: 5’-CCTTTTCAATCTGAGCTCTTCTTTCT) (Eurogentec, Seraing, Belgium), and a TaqMan probe (FAM reporter and TAMRA quencher; 5’-CACTGGAGCTGCGGTCGCTGCT)(Applied Biosystems). Quantification relative to HLA-G-positive choriocarcinoma JEG-3 cells (assigned a value of 1) was determined using the ∆∆Ct method [23]. Standard RT-PCR was carried out on 0.5 µg cDNA with pan HLA-G specific primers G.257F (forward: 5’-GGAAGAGGAGACACGGAACA; exon 2) / G.1004R (reverse: 5’-CCTTTTCAATCTGAGCTCTTCTTT; exon 5/exon 6) and HLA-G5 specific primers G.526F (forward: 5’-CCAATGTGGCTGAACAAAGG; exon 3) / G.i4b (reverse: 5’-AAAGGAGGTGAAGGTGAGGG; intron 4) for 35 cycles, and β-actin as an internal control (forward: 5’-ATCTGGCACCACACCTTCTACAATGAGCTGCG; reverse:5’ -CGTCATACTCCTGCTTGCTGATCCACATCTGC) [24] for 16 cycles. After amplification, PCR products were separated on a 1.5% agarose gel containing Ethidium Bromide.

### 2.10. Statistical Analysis

Statistical tests and graphic representations were performed with GraphPad Prism 6 (GraphPad Software, San Diego, CA, USA). All the data are presented as mean ± standard deviation (SD) of independent experiments with cells from different donors that were pooled. Significant statistical differences were calculated using one- or two-way ANOVA (analysis of variance). A *p*-value less than 0.05 was considered significant for the ANOVA. If significance existed, a post-hoc analysis was performed using the Bonferonni post-tests to evaluate significance for all experiments. For mixed lymphocyte reaction (MLR) results, the Mann–Whitney test was used to determine the significance between two groups. The difference was considered significant when the *p*-value was below 0.05.

## 3. Results

### 3.1. Cell Quality Control

**Figure 1:**
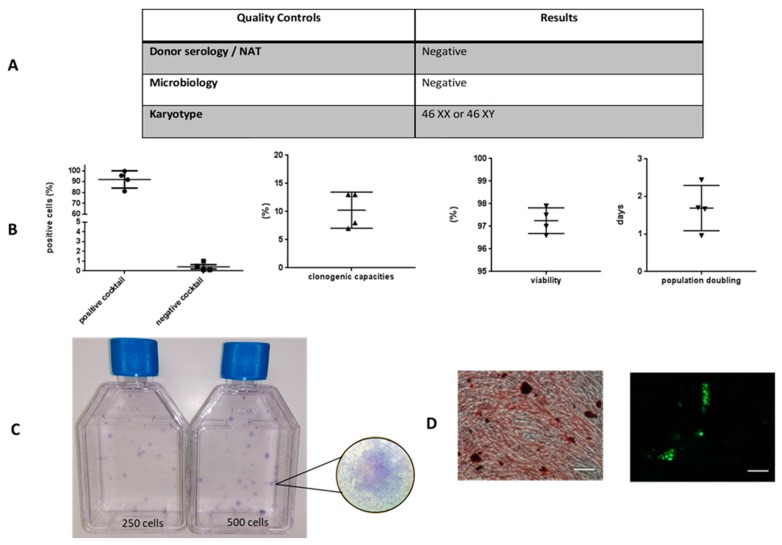
Characterization of Wharton jelly-mesenchymal stem/ stromal cells (WJ-MSCs) at the end of Good Manufacturing Practice (GMP) expansion (passage 3). Serology, Nucleic acid test (NAT), microbiology and karyotype (**A**) were determined for all cords. Graphics represent positive (Cluster of Differentiation 90 (CD), CD105, CD73, and CD44) and negative (CD45, CD34, CD11B, CD19, and Human Leukocyte Antigen (HLA) DR) cocktails, percentage of clonogenic capacities, viability and population doubling in days (**B**). Representative clonogenic capacity (**C**) and osteogenic and adipogenic differentiation are presented in (**D**). Scale bars correspond to 100 µm. Data are expressed as mean ± SD (*N* = 4 independent cords).

We first characterized the studied WJ-MSCs. The average cell population doubling time in P3 cell cultures was 1.69 ± 0.3 days (Figure 1), when seeding density of 1000 cells/cm^2^ was applied. The viability was higher than 97% at the end of GMP expansion. Immunophenotypic characterization of cells using flow cytometry demonstrated that MSCs remained negative for CD14, CD34, HLA-DR, CD11b, and CD19 and positive for CD73, CD90, CD105, and CD44, with expression levels above 97%. Moreover, the isolated UC-derived cell populations exhibited clonogenic capacities and a potential to differentiate along osteogenic lineage and, less extended, adipogenic lineage. Moreover, MSC productions presented no bacterial contamination and a normal karyotype (Figure 1).

### 3.2. Chondrocyte Differentiation

**Figure 2:**
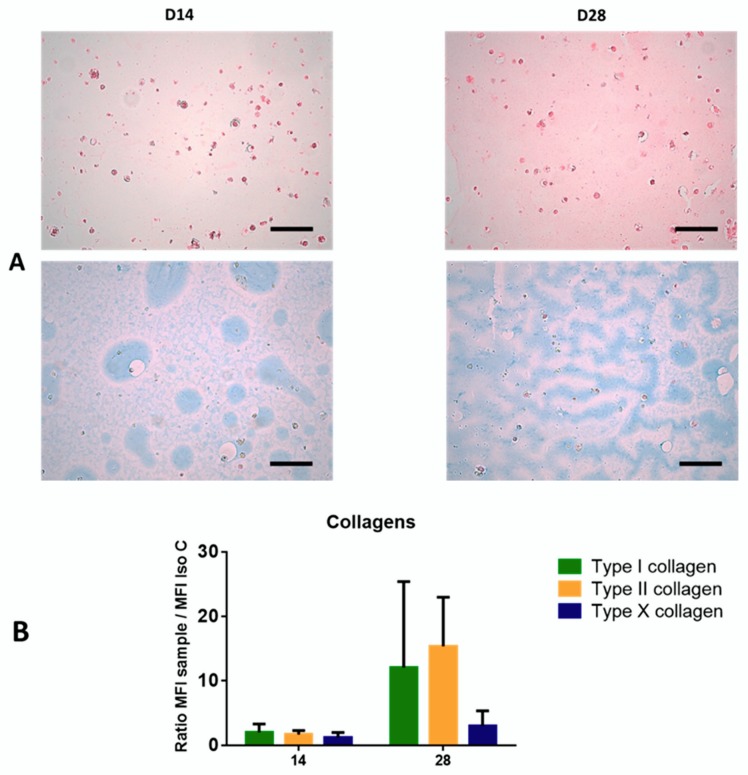
Chondogenic differentiation represented by matrix synthesis (**A**) and pericellular collagen expression (**B**). The synthetized matrix by chondro-MSCs seeded in alginate (Alg)/hyaluronic acid (HA) hydrogels was studied in histological sections (**A**) through the detection of collagen fibers (sirius red staining) and proteoglycans (alcian blue staining). Histological sections were obtained on Day 14 (D14) and Day 28 (D28) of chondrocyte differentiation. Scale bars correspond to 100 µm. Pericellular collagens (type I, II and X collagen) around chondro-MSCs were analyzed by flow cytometry in ratio of mean fluorescence intensity (MFI) of the sample compared with the isotype control (Iso C) (**B**) at different time points of cell culture (D14 and D28). Biomaterial dissolution was performed before indirect staining. Data are expressed as mean ± SD (*N* = 3 independent cords). No statistically significant difference was observed.

Chondrocyte differentiation was evaluated using Alg-based hydrogel during 28 days through collagen and proteoglycan synthesis. After 28 days of culture in Alg/HA hydrogel, a beginning of matrix synthesis was observed around cells on the histological sections. This was confirmed both by a sirius red stain to detect total collagen and an alcian blue stain to detect total proteoglycan (Figure 2A). More precisely, we showed that MSCs seeded in Alg/HA scaffolds expressed mainly type II collagen on D28 of the differentiation but also type I (Figure 2B). It appears that type X collagen associated with hypertrophyc chondrocyte was not expressed on D14 or on D28 of chondrogenic differentiation.

### 3.3. Viability

**Figure 3:**
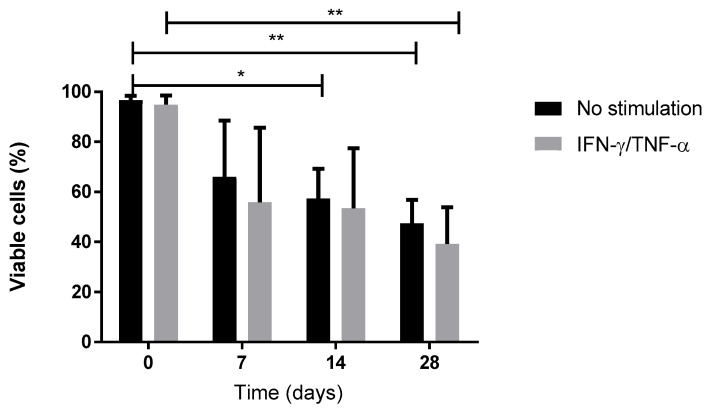
Cell viability for stimulated (IFN-γ and TNF-α) or unstimulated MSCs (day 0) and chondro-MSCs during 28 days of culture in Alg/HA hydrogels. Data are expressed as mean ± SD (*N* = 4 independent cords). * *p* < 0.05 and ** *p* < 0.01.

Above all, we checked cell viability both during 3D culture into Alg/HA hydrogel and also when cells were stimulated with IFN-γ and TNF-α. These two conditions may have had an impact not only on cellular behavior but also on cell viability. We observed no significant difference between cells seeded in biomaterial with or without stimulation during the differentiation, although cells seeded in biomaterial appear to be less viable than monolayer cells (Figure 3).

Since cell viability was investigated by flow cytometry with an antibody that reacts with amines (LIVE/DEAD kit, Thermo Fisher Scientific, Waltham, MA, USA), it was possible to detect cell viability in all analyzed samples. Results in flow cytometry were obtained using a gate only on viable cells, with this method for all results.

### 3.4. Immune phenotype

#### 3.4.1. Immune Synapse Markers

**Figure 4:**
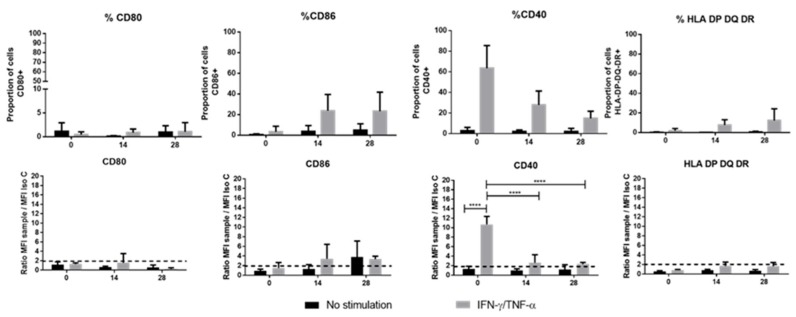
Main molecule expression related to the immunological synapse. These molecules (CD80, 86, 40 and Human Leucocyte Antigen (HLA)-DP-DQ-DR) were studied on Day 0 (monolayer) and during chondrogenic differentiation (day 14 and 28) in two conditions (with Interferon-γ (IFN-γ) and Tumor Necrosis Factor-α (TNF-α) stimulation (grey color) or without stimulation (black color). Results were obtained by flow cytometry and calculated in proportion of positive cell for each marker (up results) and ratio of mean fluorescence intensity of the sample compared with the isotype control (down results). The dotted lines represent the expression level for each molecule ratio higher than 2 between sample and isotypic control (down results). Data are expressed as mean ± SD (*N* = 3 independent cords minimum), **** *p* < 0.0001.

The activation of the immune system in an allogeneic context (T lymphocyte activation) is associated with the expression of co-stimulatory molecules: CD80, CD86, and CD40, and with the expression of MHC-II. First of all, it should be noted that the only significant variations observed were not in the proportion of positive cells for a marker but rather in the mean fluorescence intensity on the cell surface, reflecting the number of receptors expressed per cell (Figure 4). No expression of MHC-II molecules was observed (HLA DP-DQ-DR), regardless of the culture conditions or the culture duration. Among the costimulation molecules, CD80 was never expressed and CD86 was detected only on cells stimulated with IFN-γ and TNF-α throughout the entire process of differentiation, and on D28 in the absence of stimulation. Finally, the CD40 molecule was highly expressed in stimulated monolayer cells and only slightly on stimulated chondro-MSCs on D14 and D28, significantly only as a ratio of mean fluorescence intensity (*p* < 0.0001) (Figure 4).

#### 3.4.2. Immunomodulatory Factors

Several molecules contribute to immune properties, especially molecules involved in immunomodulation. PD-L1 expression decreased between D0 and D28 regardless of culture condition, but remained expressed throughout these cultures only with IFN-γ and TNF-α stimulation. A significant difference was observed between the stimulated and non-stimulated cells on D14 of the differentiation only in ratio of mean fluorescence intensity.

**Figure 5:**
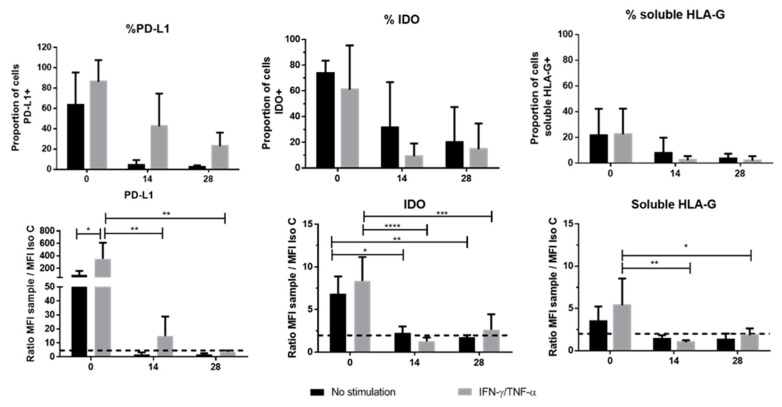
Immune checkpoint molecules involved in cell immunomodulation. Membrane Programmed death-ligand 1 (PD-L1), Indoleamine 2,3 dioxygenase (IDO) enzyme and soluble Human Leucocyte Antigen G (HLA-G) were studied on Day 0 (monolayer) and during chondrogenic differentiation (Day 14 and 28) in two conditions (with stimulation (grey color) or without stimulation (black color). Results were obtained by flow cytometry and calculated calculated in proportion of positive cell for each marker (up results) and in ratio of mean fluorescence intensity of the sample compared with the isotypic control (down results). The dotted lines represent the expression level for each molecule (ratio higher than 2 between sample and isotypic control). Data are expressed as mean ± SD (*N* = 3 independent cords minimum), * *p* < 0.05; ** *p* < 0.01; *** *p* < 0,001; **** *p* < 0.0001.

We observed a significant difference between WJ-MSCs and chondro-MSCs for IDO and soluble HLA-G, but no major difference was observed between stimulated and non-stimulated cells stimulated only in ratio of mean fluorescence intensity (Figure 5). However, no expression for membrane and soluble HLA-G1 was observed.

**Figure 6:**
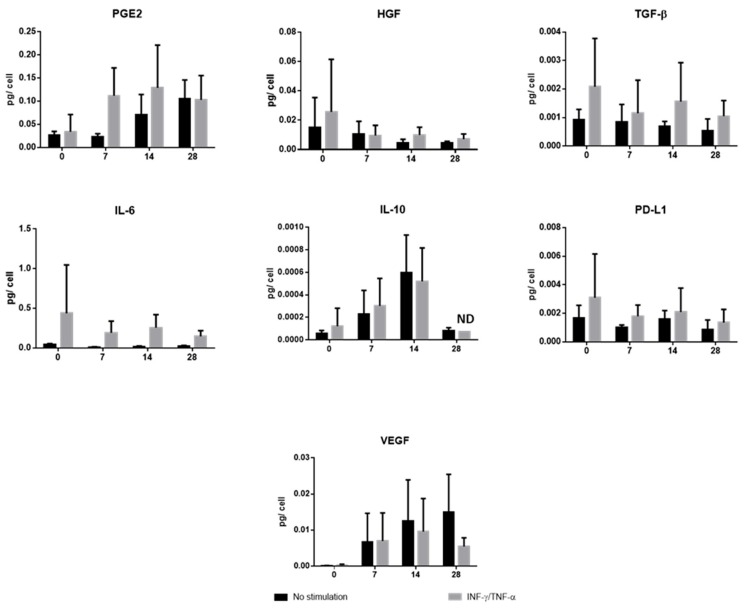
Cytokines (Interleukin 6 (IL-6) and Interleukin 10 (IL-10)), growth factors (hepatocyte growth factor (HGF), transforming growth factor beta 1 (TGF-β1) and vascular endothelial growth factor (VEGF)) and other molecules (Prostaglandin E2 (PGE2) and soluble PD-L1) secreted by mesenchymal stem/stromal cells (MSCs) on Day 0 (monolayer) and during chondrogenic differentiation (Day 7, 14, and 28) in two conditions (with stimulation (grey color) or without stimulation (black color). Results were obtained with Enzyme-Linked Immunosorbent Assay (ELISA) and were calculated in picogram per cell. Data are expressed as mean ± SD (*N* = 3 independent cords minimum); ND means non-detectable for the other 2 cords.

Immunomodulatory properties are also found through a contact independent pathway with other secretions of soluble factors. Seven molecules were dosed in MSCs supernatant: PGE2, HGF, TGF-β1, IL-6, IL-10, PD-L1 and VEGF. We observed that each molecule was expressed, especially when cells were stimulated with INF-γ and TNF-α, except for IL-10 and VEGF. Throughout the chondrocytic differentiation soluble factors were produced with no differences from the monolayer, except for HGF, which had a slight decrease after monolayer seeding and for IL-10 on Day 28 (Figure 6).

### 3.5. Immune Functional Properties of WJ-MSCs and Chondro-MSCs

WJ-MSCs are thought to have a high potential in therapies aimed at repairing cartilage defects from various etiologies. In this context, our present work entailed assessing, from an immunological perspective, if (i) allogeneic WJ-MSCs could be used without a risk of rejection instead of autologous MSCs from bone marrow or adipose tissue and if (ii) chondrogenic differentiation of WJ-MSCs affects their immunological properties. For this purpose, we evaluated both immunogenicity and immunomodulation properties of WJ-MSCs (on Day 0) and chondro-MSCs (on D28 along the chondro-differentiation process) in HLA-mismatched settings.

**Figure 7:**
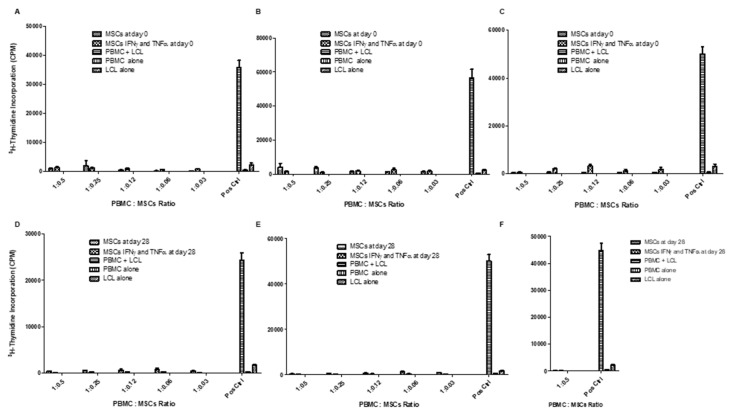
WJ-MSCs (Day 0) and chondro-MSCs (Day 28) showed hypo-immunogenicity even with IFN-γ and TNF-α pre-treatment. Distinct WJ-MSCs (**A–C**) and chondro-MSCs (**D–F**) primed with pro-inflammatory cytokines, or not, were used as stimulators facing HLA-mismatched PBMC at various PBMC: MSC ratios. Pre-irradiated LCL cells were used as a positive control (Pos Ctrl) to stimulate PBMC alloproliferation (PBMC + LCL). Data are expressed as mean CPM ± SD. CPM—count per minute; LCL—lymphoblastoid cell line; PBMC—peripheral blood mononuclear cell.

To assess the immunogenicity of both WJ-MSCs and chondro-MSCs, we studied their ability to be recognized as allogeneic-stimulating cells by HLA-mismatched T cells present in the responding PBMC. The MLR based on the use of HLA class II+ LCL stimulating cells facing the HLA-mismatched PBMC constituted the positive control. Results showed no T cell alloproliferation in response to various amounts of WJ-MSCs (Figure 7A–C) or chondro-MSCs (Figure 7D–F), even at high doses (50,000 cells corresponding to 1:0.5 PBMC:MSC ratio) and after licensing with IFN-γ and TNF-α. Figure 6 shows raw data obtained with three distinct representative PBMC:WJ-MSCs (A–C) or chondro-MSCs (D–F) allogeneic combinations. In Figure 6F, only the highest PBMC:MSC ratio (1:0.5) was tested due to limited chondro-MSCs cell numbers. Although inter-individual variability was observed, similar results were obtained using 7 distinct WJ-MSCs with PBMC from three distinct donors.

**Figure 8:**
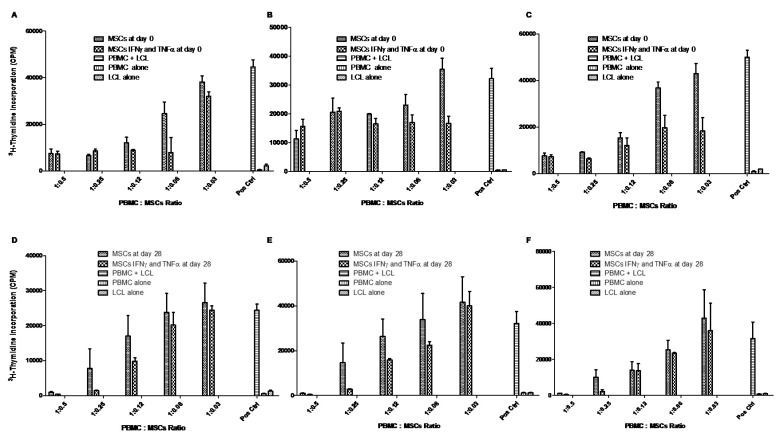
WJ-MSCs (Day 0) and chondro-MSCs (Day 28) showed immunomodulatory properties in a dose dependent manner. Distinct WJ-MSCs (**A**–**C**) and chondro-MSCs (**D**–**F**) primed with pro-inflammatory cytokines, or not, were added as third-party cells at various ratios into an MLR using PBMC as HLA-mismatched responder T cells towards irradiated LCL used as stimulating cells. Control without MSCs correspond to PBMC+LCL (Pos Ctrl). Data are expressed as mean CPM ± SD. CPM—count per minute; MLR—mixed lymphocyte reaction; LCL—lymphoblastoid cell line; PBMC—peripheral blood mononuclear cell.

To examine the immunomodulatory properties of WJ-MSCs and chondro-MSCs, we studied their ability to affect T cell alloproliferation as third-party cells in a classic MLR (immunosuppression assay). Our data revealed that WJ-MSCs and chondro-MSCs inhibit T cell alloproliferation in a dose-dependent manner. Figure 8 displays raw data obtained with three representative PBMC:LCL:WJ-MSC (A–C) or chondro-MSC (D–F) allogeneic combinations, respectively. When considering the T cell alloproliferation toward LCL to be 100%, the inhibition exerted by WJ-MSCs or chondro-MSCs was 50% (*p* < 0.0001) and 97% (*p* = 0.005), respectively, at a PBMC:MSC ratio of 1:0.5; 54% (*p* < 0.0001) and 66% (*p* = 0.005), respectively, at a PBMC:MSC ratio of 1:0.25; 63% (*p* < 0.0001) and 32% (*p* = 0.045), respectively, at a PBMC:MSC ratio of 1:0.12; and 43% (*p* < 0.0001) at a PBMC:MSC ratio of 1:0.06. Notably, the immunomodulatory potential of chondro-MSCs was higher after licensing with INF-γ and TNF-α (98% (*p* = 0.0005), 93% (*p* = 0.005), and 60% (*p* = 0.005) at a PBMC:MSC ratio of 1:0.5, 1:0.25 and 1:0.12 respectively). These results were obtained with three distinct cords and three distinct PBMCs. It is important to note that we observed individual variability among the distinct MSCs and PBMCs tested.

### 3.6. HLA-G Transcriptional Activity in WJ-MSC Upregulated during Chondrogenic Differentiation and Boosted by Inflammatory Environment 

**Figure 9:**
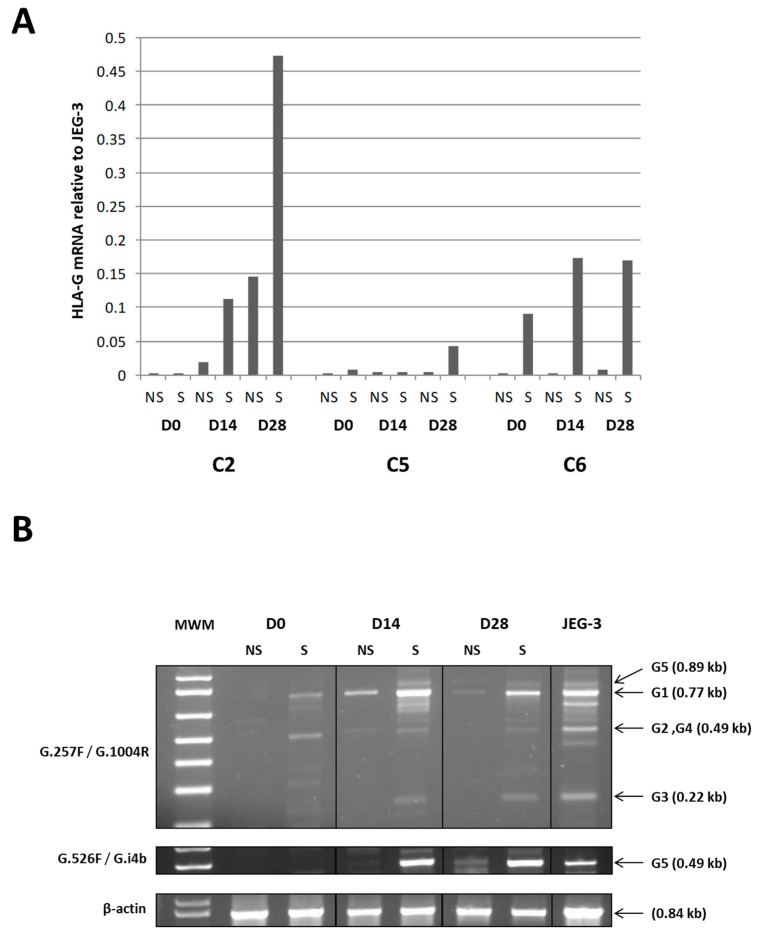
Real-time RT-PCR analysis. (**A**) showing relative quantities of HLA-G transcripts in WJ-MSC from three umbilical cords (C2, C5, C6) at the end of the cell expansion, Day 0 (D0), and on Day 14 (D14) and Day 28 (D28) during chondrogenic differentiation in Alg/HA hydrogel, in the absence (NS) or presence (S) of Interferon γ (IFN-γ) and Tumor Necrosis Factor α (TNF-α). The amount of HLA-G transcripts in JEG-3 cells was assigned a value of 1. No variation of GAPDH mRNA levels were observed considering the conditions used and chondrogenic differentiation. Representative standard RT-PCR analysis (**B**) was performed with the C6 umbilical cord on D0, D14, and D28, and JEG-3 cells targeting least alternative transcripts HLA-G1,-G2,-G3/G4 and G5 with primer set G.257F / G.1004R and HLA-G5 alone with primer set G.526F/G.i4b. β-actin amplification was used as internal control. MWM—molecular weight marker; NS and S-WJ-MSCs not stimulated or stimulated, respectively, with IFN-γ and TNF-α. PCR products obtained with each primer set were collected from the same agarose gel to assemble the picture.

Considering the key role of HLA-G in immunoregulatory processes [18], we further examined mechanisms regulating its protein expression in WJ-MSC expansion (Day 0) and during chondrogenic differentiation (D14 and D28) (Figure 4) by analyzing HLA-G transcriptional activity. We performed real-time and standard RT-PCR targeting HLA-G transcripts in cells derived from three umbilical cords treated or not treated with IFN-γ and TNF-α. We observed the absence or very low levels of HLA-G transcriptional activity in WJ-MSCs on Day 0 and a weak upregulation upon stimulation. The amount of HLA-G transcripts increased during chondrogenic differentiation, particularly in the presence of an inflammatory microenvironment (Figure 9). Notably, the increase in transcriptional activity varied according to the umbilical cord; with low activity for C5 and high activity for C2 (Figure 9A). Finally, all alternative transcripts were involved, including HLA-G5, which encodes the soluble HLA-G molecule.

## 4. Discussion

Regenerative medicine emerged a few years ago for cartilage reparation and has become a promising therapeutic alternative especially using adult MSCs in combination with innovative scaffolds. However, as BM-MSC capacities have been shown to decrease with the donors’ age [25], we investigated Wharton’s jelly from the umbilical cord as an alternative source of MSCs. WJ-MSCs seem more immature from an immunological point of view, have a higher proliferation potential, and can differentiate into a higher number of tissues. The strategy will be to implement WJ-MSCs seeded in a 3D scaffold in an allogeneic context. However, the maintenance of their immunomodulatory properties and their paracrine activity during chondrogenic differentiation remain unknown. The aim of our work was, thus, to investigate, at the same time, the differentiation of WJ-MSCs for the treatment of cartilage defects and the persistence of their immunomodulatory effects on the immune system. We propose an innovative model of cartilage engineering using WJ-MSCs produced as an Advanced Therapy Medicinal Product (ATMP) made from only clinical-grade reagents or synthetic reagents with no animal protein. This product was extemporaneously embedded into an Alg/HA layer guiding cells to chondrogenic differentiation. We demonstrated that WJ-MSCs seeded in this biomaterial were engaged in the process of chondrocyte differentiation from Day 14, while maintaining their immunomodulatory properties.

Above all, we showed that at the end of monolayer expansion, the obtained cells could be considered mesenchymal stromal/stem cells in accordance with the International Society for Cell and Gene Therapy (ISCT) [5], due to their stemness, clonogenicity, surface marker expression, infectious markers, microbiology, and karyotype. In our GMP condition, WJ-MSCs displayed robust proliferation abilities. Self-renewal and high proliferation capacity are known to be essential for MSCs to maintain stemness characteristics during in vitro culture. Regarding differentiation, despite appropriate culture medium for adipogenic differentiation, WJ-MSCs weakly differentiated into adipocytes, as reported by Reppel et al. [11]. It is well-known that adipogenic induction could be regulated by Insulin-like growth factor binding protein 2 (IGFBP2) expression. Recently Wang et al. showed that, in WJ-MSCs, IGFBP2 expression was negatively regulated by BCL6 corepressor (BCOR) [26], which could partially explain the difficulty of cells to engage in this differentiation. Our results also showed the multilineage differentiation potential of WJ-MSCs, particularly for osteogenesis and chondrogenesis. Given our goal, we initiated chondrocyte differentiation into Alg/HA hydrogels. On Day 28, MSCs underwent chondrocyte differentiation attested by the beginning of collagen synthesis, especially type II collagens, which could reflect matrix production associated with hyaline cartilage. Type I collagen seems to be highly expressed from the beginning of 3D culture, certainly due to a physiological expression of type I collagen by MSCs in the umbilical cord matrix as shown by Margossian et al. [27]. However, the presence of type X collagen expressed by hypertrophic chondrocytes was quite low, indicating a chondrocyte-limited differentiation. Our results confirm that the composition of the hydrogel alone can guide WJ-MSCs in a process of chondrocyte differentiation. These results are in agreement with previous studies [11,22,28] but are the first obtained from a study that follows good manufacturing practices with the aim of using the biomaterial and chondro-MSCs derived from Wharton’s jelly as an ATMP. Moreover, during cell differentiation, we showed a decrease in cell viability in biomaterials with respect to monolayer. We have previously described this evolution when cells were encapsulated in alginate enriched in hyaluronic acid and have shown that mechanical environment influences cell behavior and viability [22]. However, we observed that viable cells remain functional and are not only able to induce chondrocyte differentiation but also inhibit T cell alloproliferation.

Regarding the functional aspect of MSCs, we investigated whether WJ-MSCs (control cells on Day 0) and chondro-MSCs were able to induce an allogeneic stimulation. Thus, in the presence of PBMC from different-HLA healthy donors or LCL, both WJ-MSCs and chondro-MSCs were non-immunogenic and present immunomodulatory properties. This was observed mainly when cells were primed with IFN-γ and TNF-α, as previously reported with MSCs and chondro-MSCs from bone marrow and adipose tissue [29]. Indeed, it was once again demonstrated that IFN-γ and TNF-α priming is favorable for enhanced immunoregulatory properties, as shown by Prasanna et al. [30].

However, the immunoregulatory properties of immune cells are described to be regulated through cell-to-cell contact or the paracrine effect. To know how these properties are regulated by MSCs and chondro-MSCs, both immunogenicity and immunomodulatory properties were evaluated by analyzing the expression of stimulation molecules as well as immune checkpoints and T cell alloresponse, in the presence or absence of IFN-γ and TNF-α to mimic an allogeneic context.

Indeed, in a context where a donor is different from a recipient, immune cells can be activated through the implementation of the immunological synapse [31]. This synapse involves the MHC-II (HLA-DP-DQ-DR) as well as the co-stimulation molecules (CD40, CD80 and CD86) to activate and present the antigen to T-cells. It is well-known that, after monolayer expansion, MSCs do express neither MHC-II nor the previously mentioned co-stimulatory molecules [31]. In contrast, it has been reported that primed MSCs with IFN-γ and/or TNF-α enhance the expression of MHC-II but do not seem to increase the levels of CD80 and CD86 [32]. In our study, unlike that of Ryan et al., who report an expression of MHC-II on BM-MSCs differentiated in chondrocytes in an alginate hydrogel [33], we did not observe any expression of MHC-II or CD80 by either WJ-MSCs or chondro-MSCs, whether cells were primed or not. Consequently, since MHC-II and co-stimulation molecules were not simultaneously present on WJ-MSCs or chondro-MSCs, the partial immunological synapse formed could not sustain the activation of T cells. These observed differences from the study of Ryan et al. may be due to the cell source as WJ-MSCs are described to have stronger immune capacities than BM-MSCs [34].

We also studied the expression of other checkpoint molecules, such as IDO, HLA-G and PD-L1. The expression level of IDO at the end of the 3D culture suggested that this enzyme could play a minor role in the immune properties of chondro-MSC. Taking into consideration the specific involvement of HLA-G in MSC-mediated immunomodulation, we focused our attention on the mechanisms involved in HLA-G expression levels [17]. First, high variation in the levels of HLA-G transcripts, likely driven by HLA-G polymorphism, was observed among the various umbilical cords [35]. Second, we found that global HLA-G transcription including *HLA-G1* and *HLA-G5* transcripts in WJ-MSCs and chondro-MSCs may have been stimulated by INF-γ and TNF-α treatment, which is consistent with previously published results of MSCs derived from bone marrow and adipose tissue [29]. Interestingly HLA-G1 protein expression was not detected at the cell surface of either WJ-MSCs or chondro-MSCs in the presence or absence of treatment, suggesting that other membrane-bound isoforms may be upregulated at the cell surface and, thus, could participate in the immunomodulatory functions. In addition, we found that *HLA-G5* transcripts may be absent in WJ-MSCs even upon stimulation, while they were upregulated in chondro-MSCs. This result suggests that soluble HLA-G expression detected in WJ-MSCs involves other alternative transcripts. Discrepant results regarding HLA-G gene transcription and protein expression may be explained by post-transcriptional regulatory mechanisms, such as miRNAs that control HLA-G protein expression. 

The receptor programmed death-1 (PD-1) was expressed on the cell surface of different immune cells like activated T and B cells. Interaction of PD-1 with its ligands, PD-L1/B7-H1 and PD-L2/B7-DC, provides an inhibitory signal in regulating cellular activation and proliferation. After 28 days of culture, the membrane-bound PD-L1 expression was extremely low, suggesting that the chondro-MSCs did not inhibit T cell proliferation this way. However, like Davies et al. [20], we detected soluble PD-1 ligands not only in culture media containing WJ-MSCs but also in culture media after 28 days of 3D culture, suggesting that the suppression of T cell activation could occur through the interaction of soluble PD-1 ligands secreted by WJ-MSCs and chondro-MSCs.

These results, therefore, suggest that the regulation of MSCs depends first and foremost on their secretory activity. To further explore the evolution of paracrine activity during cell differentiation, we found that, in addition to soluble PD-L1, unprimed chondro-MSCs showed the ability to secrete immunomodulatory molecules, sometimes in the same proportions as WJ-MSCs, at the end of P3 (TGF-β1, IL-6), and sometimes cell differentiation allowed better secretion than WJ-MSCs (PGE2, IL-10 and VEGF). In addition, priming with INF-γ and TNF-α allowed better secretion of IL-6 and TGF-β by both WJ-MSCs and chondro-MSCs. As Zhao et al. [13] explained, these molecules specifically contribute to the generation of an immunosuppressive environment. Thus, TGF-β, PGE2 and IL-10 would induce regulatory T lymphocytes. IL-6 would inhibit the differentiation and maturation of dendritic cells. Finally, HGF would have repair characteristics and VEGF would have vascularization and proliferation properties [19,31]. Lastly, the ability of chondro-MSCs to release anti-inflammatory factors like PGE2 and IL-10 could attenuate the inflammatory environment of the joint [36].

## 5. Conclusions

In this study, mesenchymal stem/stromal cells once again emerged as a potential for cell therapy [37], especially MSCs derived from human umbilical cords [38]. The results showed that 3D culture modulates the immunomodulatory phenotype and/or the paracrine activity of chondro-MSCs. Here, we have shown a reduced immunomodulatory phenotype but, nevertheless, a secretion that seems to be maintained. We reported that WJ-MSCs and chondro-MSCs were able to decrease the activation of immune cells as well as create an immunomodulatory environment that is promising for implementation in an allogeneic context. If further in vivo studies performed in pre-clinical models confirm these encouraging results, it will pave the way to a universal allogeneic therapy of cartilage defects.
